# Level of Disability after Total Hip Replacement in Patients with Some *COMT* Gene Polymorphism

**DOI:** 10.3390/jcm12247652

**Published:** 2023-12-13

**Authors:** Alina Jurewicz, Violetta Dziedziejko, Monika Rać, Marta Białecka, Krzysztof Safranow, Mateusz Kurzawski, Damian Malinowski, Mateusz Bosiacki, Katarzyna Leźnicka, Andrzej Bohatyrewicz, Monika Białecka, Marek Droździk, Anna Machoy-Mokrzyńska

**Affiliations:** 1 Department of Specialistic Nursing, Pomeranian Medical University, Żołnierska St. 48, 71-210 Szczecin, Poland; alina.jurewicz@pum.edu.pl; 2 Department of Biochemistry and Medical Chemistry, Pomeranian Medical University, Al. Powstańców Wielkopolskich 72 St., 70-111 Szczecin, Poland; viola@pum.edu.pl (V.D.); chrissaf@mp.pl (K.S.); 3 Department of Pharmacology, Pomeranian Medical University, Al. Powstańców Wielkopolskich 72 St., 70-111 Szczecin, Poland; marta.bialecka@post.pl (M.B.); mateusz.kurzawski@pum.edu.pl (M.K.); damian.malinowski@pum.edu.pl (D.M.); marek.drozdzik@pum.edu.pl (M.D.); 4 Department of Functional Diagnostics and Physical Medicine, Pomeranian Medical University in Szczecin, Żołnierska St. 54, 71-210 Szczecin, Poland; bosiacki.m@gmail.com; 5 Faculty of Physical Education, Gdansk University of Physical Education and Sport, K. Górskiego St. 1, 80-336 Gdansk, Poland; katarzyna.leznicka@awf.gda.pl; 6 Department of Orthopedics, Traumatology and Musculoskeletal Oncology, Pomeranian Medical University, Unii Lubelskiej St. 1, 71-252 Szczecin, Poland; andrzej.bohatyrewicz@pum.edu.pl

**Keywords:** total hip replacement, disability, *COMT* gene polymorphism

## Abstract

Background: The *COMT* gene encodes the enzyme catechol-O-methyltransferase, which is a key modulator of dopaminergic and adrenergic neurotransmission. Hip osteoarthritis is accompanied by reduced mobility and some level of disability. In our study, we analyzed the association between some *COMT* gene polymorphisms and reduced mobility in patients after total hip replacement (THR). Methods: The operative procedures were performed on 195 patients with symptomatic and radiologically advanced hip osteoarthritis. In the postoperative follow-up, we assessed hip function with the Harris Hip Score (HHS) and the degree of disability with the Oswestry Disability Index (ODI). These procedures were repeated three times at defined intervals (one week, six weeks, and six months) after the total hip replacement. Genomic DNA was extracted from peripheral blood. SNPs in the *COMT* genes *rs4680:A>G*, rs6269:*A>G*, rs4633:*C>T*, and rs4818:*C>G* were genotyped. Results: Our findings suggest an association between COMT gene variability and the level of disability measured by the Oswestry Disability Index (ODI) in patients after total hip replacement (THR). Conclusions: A higher number of *COMT* G alleles (rs4818) is an independent factor in a significant reduction in disability degree at both one week and six months after total hip replacement (THR), regardless of age or gender.

## 1. Introduction

Degenerative hip joint disease is considered one of the relatively common musculoskeletal diseases. It is usually accompanied by functional impairment and changes in the structure of joints, which are likely to lead to disability. The hip joint, being a synovial joint, is predisposed to the development of signs of inflammation. Fantoni et al. [1] have observed that, in patients with hip osteoarthritis, an increase in collagen type I along with a reduction in collagen type III and hyaluronan could lead to fascial stiffening, which could alter the fascial mechanics and be linked to the development and symptoms of the disease. However, the etiology of this disease is multifactorial, involving constitutional factors, such as age, gender, and obesity, along with genetic factors, as well as local mechanical factors, such as trauma, professional activity-related or sports-related excessive strains, abnormal alignment of the joint axis, etc. Significant impairment in joint mobility impacts patients’ quality of life and increases the risk of other diseases reported by patients [2].

Population ageing in developed countries will inevitably increase the need for hip replacement surgery. Recently, more attention has been paid to genetic factors like single nucleotide polymorphisms (SNPs) as genetic variations that may contribute to individual susceptibilities to certain pathological conditions after THA. Various gene SNPs were reported as markers of increased risk. Henkel et al. [3] identified, in Genome-Wide Association Studies (GWAS), sequence variants associated with hip osteoarthritis, such as missense variants in *COL11A1* (encoding collagen type XI), *SMO* (encoding smoothened protein), *IL1* (encoding interleukin 1), and *SLC39A8* (encoding metal cation transporter) genes, but also intronic variants in *P3H2* (encoding prolyl 3-hydroxylase 2), *RNF130* (encoding E3 ubiquitin ligase), *PIEZO1* (encoding cation channel), *PLCL2* (encoding phospholipase C), and variants of UTR fragments in *SHBG* (encoding sex hormone binding globulin) and *NACA2* (encoding isoform of Na^+^/Ca^2+^ plasma membrane) genes. Also, a variable number of tandem repeats in intron 2 of the *IL1* gene (rs2234663) was strongly associated with aseptic non-mechanically caused symptomatic hip arthroplasty [4]. In the next study [5], was identified a *SNP* of the *KPG* gene encoding kidney gamma-carboxyglutamate—proteins associated with inflammation and macrophage activation. This SNP was associated with significantly increased odds of failure and revision for hip replacements. Another study [6] demonstrated that *MMP-1* (encoding matrix metalloproteinase) rs5854 C/T polymorphism was associated with an increased risk of early failure of THA, such as aseptic implant loosening. Evaluation of the *MMP-1* rs5854 SNP may be a useful marker in the diagnosis and prevention of its risk. Several of the identified genes are involved in osteoarthritis-relevant processes, for example, autophagy, osteogenesis, chondrogenesis, mechanotransduction, and angiogenesis [7].

The *COMT* gene encodes the enzyme catechol-O-methyltransferase, which metabolizes dopamine, epinephrine, and norepinephrine and is a key modulator of dopaminergic and adrenergic neurotransmission [8]. One of the most commonly described polymorphisms within this gene is the single-nucleotide polymorphism (SNP), rs4680:*A>G*, which changes the amino acid valine to methionine at position 158 [9]. The described SNP results in different metabolic activity of the enzyme: low (COMT^L/L^), high in wild-type homozygotes (COMT^H/H^), or intermediate (COMT^H/L^). Previously published research suggested that the metabolic activity of the COMT enzyme may also depend on the gene haplotype. In terms of the haplotype, apart from the rs4680 polymorphism which was already mentioned, two silent exon polymorphisms, rs4633:*C>T* and rs4818:*C>G*, and the promoter polymorphism, rs6269:*A>G*, are also included [10,11]. Hip osteoarthritis is accompanied by a reduced functional capacity. Genetic variation in the *COMT* gene has been implicated in variable susceptibility to developing common conditions associated with disability because the metabolic activity of the COMT enzyme may also depend on the gene haplotype [12,13].

The aim of this study was to evaluate the possible association between selected *COMT* gene polymorphisms and the degree of disability in patients with degenerative hip disease treated surgically with elective total hip arthroplasty (THA).

## 2. Methods

### 2.1. Study Group Characteristics

**Figure 1:**
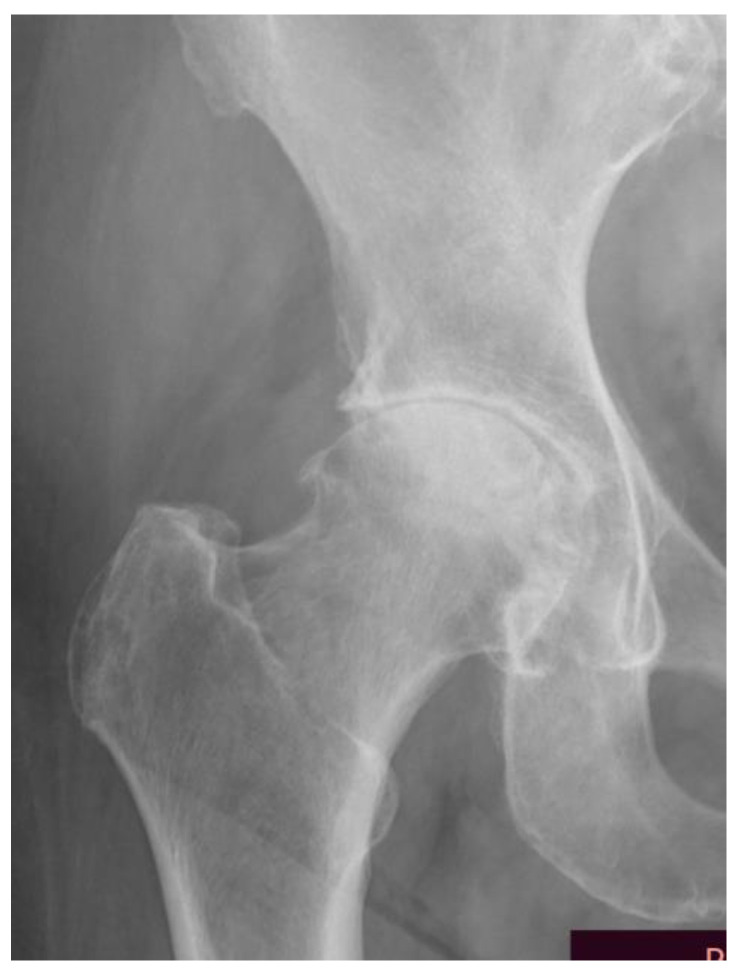
Moderate hip osteoarthritis Lawrence and Kellgren grade 3 (from the archives of author A. Bohatyrewicz).

**Figure 2:**
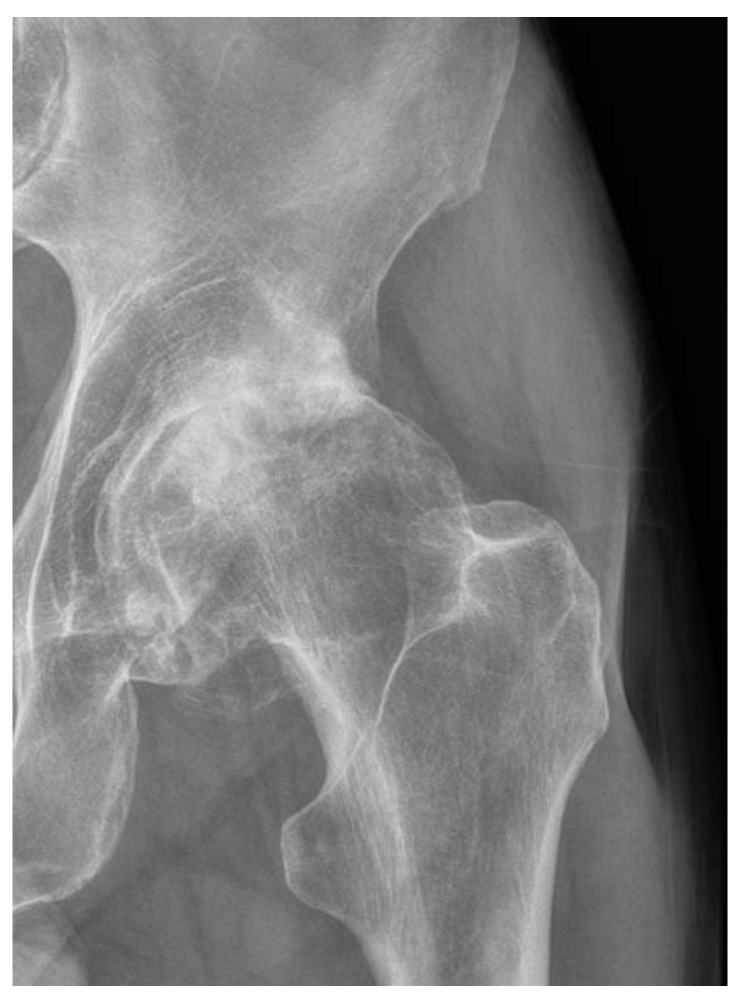
Severe hip osteoartritis Lawrence and Kellgren grade 4 (from the archives of author A. Bohatyrewicz).

**Figure 3:**
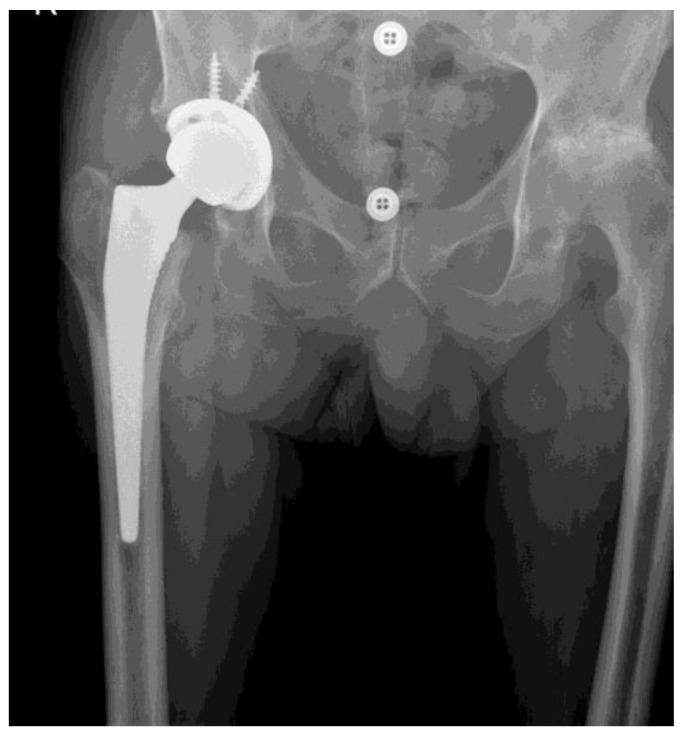
Pinnacle cup and Corail stem implants (DePuy Synthes, Warsaw, IN, USA) —radiograph after implantation—arthroplasty (from the archives of author A. Bohatyrewicz).

The group of 195 patients included for clinical observation consisted of 126 women (64.6%) and 69 men (35.4%). The mean age was 67 (±11 years), with an average age of 64 years for women and 69 years for men. The study group was recruited from the Department of Orthopedics, Traumatology, and Musculoskeletal Oncology of the Pomeranian Medical University in Szczecin, Poland. All patients included in the study gave informed written consent to participate. The study was approved by the Bioethics Committee at the Pomeranian Medical University (approval No. BN-001/6/07). Based on their clinical condition and imaging studies, the patients met the arthroplasty criteria and were qualified for THR. In order to avoid the influence of other factors on the study’s results, we used the following inclusion criteria: diagnosis of degenerative changes in the hip joints based on clinical examination and imaging—radiological examination (described as moderate or severe changes according to the Kellgren and Lawrence classification [14,15]—grade 3 and 4 (Table 1, Figure 1 and Figure 2), and age over 18 years, and exclusion criteria: osteoporosis-related hip deformations, long-standing diabetes mellitus with features of polyneuropathy, diagnosed motor-sensory polyneuropathy, mood disorders of a depressive nature (treated with thymolytic drugs), dependence on opioid analgesics and anxiolytic drugs, and patients with psychoorganic syndrome (MMSE, Mini-Mental State Examination <10 points). A pre-operation pain assessment was performed by the senior orthopedic surgeon qualifying for the operation. These data were recorded as moderate or high severity of pain (measured on the VAS scale as 4–6). Data concerning pain intensity were presented in our previous paper [13]. Patients undergoing THR received multimodal analgesic therapy according to the recommendations for postoperative pain management [16]. All operative procedures were performed in patients with symptomatic and radiologically advanced hip osteoarthritis (grades 3–4) according to the Kellgren–Lawrence classification. Inflammatory, traumatic, oncologic, and septic indications were excluded. The implant used was an uncemented Corail stem and Pinnacle acetabulum (DePuy Synthes, Warsaw, IN, USA)—Figure 3. All the procedures were performed via anterolateral approach, as described by Watson-Jones [17], by two experienced hip arthroplasty surgeons.

Assessment of the impact of degenerative hip disease on patient performance can be conducted using scales and questionnaires that are already in use in clinical practice, such as the Harris Hip Score (HHS) or the Oswestry Disability Questionnaire. The HHS is used to assess the dysfunction of the affected hip joint and the treatment outcome, most commonly total hip replacement (THR) [18]. The standard version of the HHS includes questions about daily activities, and it requires clinical examination to assess the range of motion in the hip joint. The Oswestry Disability Questionnaire is also used to assess patient performance or disability and is based on questions reflecting the ability to manage everyday life reported by a patient. The degree of disability is determined by the calculated Oswestry Disability Index (ODI) and is expressed as a percentage.

### 2.2. Assessment of Hip Joint Function

The HHS was developed to assess the result of hip surgery and is intended to evaluate various hip disabilities and treatment methods in an adult population. The areas of assessment are pain, function, absence of deformity, and range of motion. The function domain consists of reports on ability to undertake daily activities (stair use, sitting, managing shoes and socks) and gait (limp, support needed, walking distance). Deformity leads to a reduction in flexion, adduction, internal rotation, and extremity length discrepancy. The range of motion includes hip flexion-extension, adduction-abduction, and external-internal rotation. The score has a maximum of 100 points (best possible outcome), covering pain (1 item, 0–44 points), function (7 items, 0–47 points) subdivided into: daily activities (14 points) and gait (33 points), absence of deformity (1 item, 4 points), and range of motion (2 items, 5 points). The higher the HHS score, the lower the dysfunction. The HHS is widely used for evaluating the outcome after hip replacement. The HHS has also been proven appropriate to measure the outcome after other interventions, such as physical therapy and treatment of femoral neck fractures [19]. In the present study, the HHS was used in the patients three times (one week, six weeks, and six months) after THR.

### 2.3. Evaluation of the Patient’s Degree of Disability

To evaluate the level of disability, the Oswestry Disability Questionnaire was applied. Based on the Oswestry questionnaire, the Oswestry Disability Index (ODI) can be determined, which provides a self-assessed functional disability score for patients with low back pain. Four versions of the ODI are available in English [20,21]. The questionnaire is divided into ten sections, one assesses pain and the other nine evaluate the limitations regarding various daily activities. Each section is scored on a 0–5 scale, where 5 represents the greatest disability. The scores of each section are added up, multiplied by 2, and expressed as a percentage. The maximum score is 100% and expresses maximum disability. For interpretation, the ODI is subdivided into five categories: (1) 0–20%, representing minimal disability; (2) 21–40%, representing moderate disability; (3) 41–60%, representing severe disability; (4) 61–80% representing disabled patients; (5) 81–100%, representing bedbound patients or patients overestimating their symptoms. In the present study, the ODI was applied to the study patients three times (one week, six weeks, and six months) after hip arthroplasty.

### 2.4. Methods Genotyping

Genomic DNA was extracted from peripheral blood using a GeneMATRIX Quick Blood DNA Purification Kit (EURx, Gdańsk, Poland). SNPs in the *COMT* genes rs4680:*A>G*, rs6269:*A>G*, rs4633:C>T, and rs4818:*C>G* were genotyped. Pre-validated allelic discrimination TaqMan real-time PCR assays were used, with specific Assay IDs: C_25746809_50, C_2538746_1, C_2538747_20, C_2538750_10; Life Technologies, Carlsbad, CA, USA. The kit contained two primers (sense and antisense) and two allele-specific TaqMan probes, each stained with a different fluorescent dye, 6-FAM or VIC. Amplification was performed using a 7500 Fast Real-Time PCR System with incorporated SDS software v5.1 for SNP genotyping (Applied Biosystems, Foster City, CA, USA), using a TaqMan GTXpress Master Mix (Life Technologies, Carlsbad, CA, USA) [13].

### 2.5. Statistical Analysis

The Shapiro–Wilk test was used to test the normal distribution of the analyzed parameters. For each SNP, comparisons were performed between three genotype groups, between carriers and non-carriers of variant allele (dominant model) and between carriers and non-carriers of wild-type allele (recessive model). Between-group differences in measurable traits were compared using the non-parametric Kruskal–Wallis test followed by Mann–Whitney U test, with patient genotypes as the between-group variable. The linear regression model adjusted for patient age and gender was used in the multivariate analysis. *p* < 0.05 was considered statistically significant. The Statistica version 10 program (StatSoft, Inc., Tulsa, OK, USA) was used for calculations.

## 3. Results

In our study, a total of 195 patients were evaluated at three different times points (one week, six weeks, and six months) following total hip replacement to assess their degree of disability using the HHS and ODI. The results of the degree of disability in patients after hip arthroplasty are shown in Table 2. The results were averaged at defined time points. According to the HHS, patients showed satisfactory functional capacities six months after the surgery. However, based on the ODI, patients demonstrated a slight degree of disability at six weeks and six months after surgery.

The current study aimed to analyze the association between *COMT* rs6269, rs4818, and rs4680 polymorphisms with hip function assessment (according to HHS) and the degree of disability (ODI) in patients at defined time points (one week, six weeks, and six months) after hip arthroplasty. Patients with *GG* and *GA* genotypes compared to those with *AA COMT* rs6269 homozygotes, and *GG* and *CG* patients versus *CC COMT* rs4818 patients, showed significantly better hip function (according to the HHS) at one week after hip arthroplasty. However, there were no statistically significant differences between the patient genotypes regarding better hip function (according to the HHS) six weeks and six months after the surgery. Patients with the *GG* rs6269 genotype compared to those with *AA* and *GA* genotypes, and those with *GG COMT* rs4818 homozygotes compared to those with *CC* and *CG* genotypes, had a significantly lower degree of disability (according to the ODI) six months after the surgery (Table 3 and Table 4). Carriers of the *G* allele, compared to patients with the *AA* rs6269 genotype, and carriers of the *C* allele and *G* allele, compared to patients with the *CC* rs4818 genotype of the *COMT* gene, showed better hip function one week after surgery and higher functional capacities six months after surgery (Table 3 and Table 4).

The degree of disability in patients with the *GG COMT* rs4680 genotype six months after THR was statistically significantly lower than in patients with *AA* homozygotes and *GA* heterozygotes (Table 5). Compared to those with the *AA* rs4680 *COMT* genotype, the carriers of the *G* allele showed higher functional capacities six months after THR (Table 5).

The *COMT* rs4818 polymorphism was selected for multivariate analysis due to it having the most vital link to the clinical features (function and physical performance). We found that an increased number of *G* alleles (*COMT* rs4818) was an age- and gender-independent factor associated with better hip functional performance as measured by the HHS one week after THR (by 3.53 points per *G* allele) (Table 6).

A higher number of *G* alleles (rs4818) was shown to be a factor associated with a reduction in patient disability, regardless of age and gender. The degree of disability was found to be significantly reduced over the evaluated time interval (by 5.83% per *G* allele) (Table 7).

A higher number of *G* alleles (rs4818) is an age- and gender-independent factor associated with a significant reduction in the degree of patient disability over the time interval evaluated (by 5.83% per *G* allele) (Table 8).

## 4. Discussion

Our current study aimed to analyze the association between *COMT* rs6269, rs4818, and rs4680 polymorphisms and hip function assessment (according to HHS) and the degree of disability (ODI) in patients at defined time points after total hip replacement. We found that patients with *GG* and *GA* genotypes compared to those with *AA COMT* rs6269 homozygotes and *GG* and *CG* patients versus those with *CC COMT* rs4818 showed significantly better hip function at one week after hip arthroplasty. In patients with the *GG* rs6269 genotype compared to the patients with *AA* and *GA* genotypes, and in patients with *GG COMT* rs4818 homozygotes compared to patients with the other genotypes (*CC* and *CG*), there was a statistically significantly lower degree of disability expressed six months after the surgery. The carriers of the *G* allele compared to the patients with the *AA* rs6269 genotype, and those with the *C* allele and *G* allele compared to those with the *CC* rs4818 genotype of the *COMT* gene, showed better hip function one week after and higher functional capacity six months after THR. The degree of disability in patients with the *GG COMT* rs4680 genotype six months after surgery was statistically significantly lower than in those with *AA* homozygotes and *GA* heterozygotes. Compared to the *AA* rs4680 *COMT* genotype, the carriers of the *G* allele showed higher functional capacities six months after THR. We also found that an increased number of *G* alleles (*COMT* rs4818) was an age- and gender-independent factor associated with better hip functional performance, as measured by the HHS one week after hip arthroplasty. However, due to the pioneering nature of this research, we are unable to compare these results with findings from other studies.

Hip joint degeneration is often related to genetic predisposition, lifestyle, comorbidities, or natural aging processes. A structural component of the disease is based on wear and tear of the cartilage covering the articular surfaces. Therefore, in the advanced form of the disease, one of the essential elements of comprehensive treatment is the total replacement of the affected hip joint with a prosthesis. Another crucial component of the comprehensive treatment of hip osteoarthritis is rehabilitation management. Therefore, the primary criteria considered in hip arthroplasty qualification, except pain intensity, are motion range and gait, which determine the quality of life. It is critical from a clinical standpoint that the dysfunction of the affected hip can impact the neighboring kinematic chains (i.e., the spine and other joints of the lower limb) [22]. Siviero et al.’s findings demonstrated that surgery represents a valid approach to severe osteoarthritis at any age, and that a comprehensive assessment, including patient-reported symptoms and outcomes, can help with identifying risk and protective factors associated with physical function [23].

Damage to the musculoskeletal system caused by a progressive pathological process is associated with severe difficulty in performing or inability to perform basic work activities, household chores, self-care, or social roles. Therefore, it likely results in numerous instances of significant disability. The loss of functional capacity is often associated with pain, limitations in physical independence, stress, lowered mood, anxiety, depression, and low self-esteem. The surgery and efforts to restore full physical activity are conducive to developing pro-health and pro-social attitudes, facilitating interpersonal contacts, enriching the patient’s sphere of inner experiences, and creating conditions for mental rest. In everyday life, it is associated with independence and more significant opportunities for social, intellectual, and cultural enjoyment. The restoration of physical activity also directly affects intellectual potential, emotion control, and the ability to learn, concentrate, and cope with daily life. It also impacts social perception, interpersonal activity, socialization, and the quality of life [24].

Arthroplasty is an effective and widely used surgical treatment for patients with clinically and radiographically significant degenerative changes in the hip joint. For this study, we selected 195 patients with degenerative hip disease who qualified for THR at the Department of Orthopedics, Traumatology, and Musculoskeletal Oncology of the Pomeranian Medical University in Szczecin, Poland. Similar to previous researchers, we used the HHS and ODI to assess patient mobility after surgery [19,20,21,25]. Additionally, we observed an increase in postoperative pain levels after the first week, which is likely attributed to the transition to more intensive mobilization and physiotherapy at the rehabilitation unit [26]. Because pain and reduced performance are some of the factors that most reduce a patient’s quality of life, notable attention has been paid to genetic factors and the search for potential ‘pain genes’ [13]. Some of them are described in detail, especially the *COMT* gene [25] and the *OPRM1* gene (µ-opioid receptor) [27]. In our previous work, we analyzed the frequency distribution in SNPs of the *COMT* gene in patients who qualified for THR due to degenerative hip disease. For the rs6269 and rs4818 SNPs, the *G* allele was less frequent (38.7% and 38.5%, respectively). We further observed that rs4680:*A>G* was in full linkage disequilibrium (D’ = 1, r^2^ = 1) with rs4633:C>T SNP. Therefore, further analyzes were conducted for only one of the two loci (rs4680) [13].

In our study, the functional capacities of patients after hip arthroplasty were assessed one week, six weeks, and six months after surgery. Numerous studies of the early outcomes of THR have shown that patients reached a normal quality of life from 3 to 12 months after surgery [28]. Mariconda et al. conducted a multivariate analysis and showed that HHS significantly affected patient’s perceptions of their life quality. This result indicates that hip function is critical in determining the patient’s overall physical and mental condition [29]. We also used the ODI, given that the degree of disability also significantly affects patients’ quality of life, especially after surgery, as confirmed by other published studies [20,21]. Six months after the surgery, the carriers of the rare *G* allele (rs6269 and rs4818) and the *C* allele of the rs4680 *COMT* gene showed higher physical performance compared to other patients who did not carry these alleles. In a multivariate analysis, a higher number of the *G* alleles (rs4818 *COMT*) was shown to be an age- and gender-independent factor associated with a lower degree of disability as measured by the ODI six months after hip arthroplasty.

The literature contains numerous reports of genes associated with inflammatory responses that may contribute to the development of osteoarthritis. Pro-inflammatory cytokines associated with bone destruction can accumulate in the synovial fluid and lead to degenerative diseases. Published results suggest that IL17A, IL1β, and IL6 are associated with the inflammatory progression of joint pain [30,31,32]. The reduction in pain experienced by these patients probably had a significant impact on their increased physical performance, as confirmed by studies on the association between the *COMT* variant rs4680 and chronic pain syndromes such as migraine, back pain, headache, or temporomandibular joint pain (TMD) [1,2].

Our research has some limitations that need to be mentioned. First, we included non-randomized patients in terms of the etiology of hip osteoarthritis, duration of the disability, extension of the disability, structural deformities, joint contractures, concomitant diseases, and individual pain perception. All of the listed factors could potentially influence the postoperative ODI and HHS tests. However, in studies like this, any randomization is not always possible, and it should be underlined that patients’ heterogeneity is typical of hip arthroplasty candidates. Therefore, we do not assume relevant changes in study results even in much larger groups of patients. Second, there are not obligatory rehabilitation programs for arthroplasty patients in Poland, and many of them continue rehabilitation individually or even discontinue it. This might influence their disability levels. But again, we do not assume a relevant influence of different rehabilitation intensities on the large group of included patients (N = 195). The last point worth mentioning is the evident gender disparity in the recruited patients (126 women and 69 men). However, the life expectancy in Poland is much longer for females (81.9 years) compared to males (74.5 years), and this significantly influences the F/M ratio on hip arthroplasty waiting lists. On the other hand, data derived from the Global Burden of Disease Studies reveal generally higher percentages of hip arthritis in women than in men, and this disparity increases with age [33].

## 5. Conclusions

It can be stated that, probably, a higher number of *COMT G* alleles (rs4818) is an age- and gender-independent factor associated with a significant reduction in the degree of disability in patients one week and six months after total hip replacement surgery. Given the pioneering nature of this research, we cannot compare our results with the findings of other studies. The results of our study suggest an association between some *COMT* gene variability and the level of disability measured by the Oswestry Disability Index in patients after total hip replacement. This observation requires further investigation. Our findings may be used as a first step to further develop individualized therapy for patients with musculoskeletal disorders, considering genetic factors in evaluating the outcomes of ongoing treatment. It is apparent that, in patients with advanced degenerative hip disease, hip arthroplasty is the treatment of choice, combined with ap-propriate physiotherapy and pharmacotherapy. Such comprehensive management significantly enhances patients’ functional capacity, regardless of their genotype.

## Figures and Tables

**Table 1:** Kellgren and Lawrence grading scale for hip osteoarthritis.

Grade	Radiological Findings
0	Normal
1	Doubtful narrowing of joint space and possible osteophytic lipping
2	Definite osteophytes, definite narrowing of joint space
3	Moderate multiple osteophytes, definite narrowing of joint space, some sclerosis, and possible deformity of the bone contour
4	Large osteophytes, marked narrowing of joint space, severe sclerosis, and a definite deformity of bone contour

**Table 2:** Degree of disability in patients after hip arthroplasty at defined time-points.

Parameter	Mean ± SD	Median	Lower Quartile	Upper Quartile
HHS after 1 week	34.9 ± 12.7	37	26	43
HHS after 6 weeks	69.9 ± 11.8	71	64	77
HHS after 6 months	86.8 ± 11.5	90	83	95
ODI after 1 week	52.2% ± 16.1%	50	40	64
ODI after 6 weeks	18.1% ± 11.0%	16	12	22
ODI after 6 months	7.1% ± 10.2%	4	0	9

HHS—Harris Hip Score; ODI—Oswestry Disability Index.

**Table 3:** Association of *COMT* rs6269 genotype with the degree of disability in patients after THR.

Parameter	*COMT* rs6269										
	*AA* (*n* = 75)	*GA* (*n* = 89)	*GG* (*n* = 31)	*AA+GA* (*n* = 164)	*GA+GG* (*n* = 120)	K-W	*AA* vs. *GA+GG*	*AA+GA* vs. *GG*	*AA* vs. *GG*	*AA* vs. *GA*	*GA* vs. *GG*
	Mean (±SD)					p^a^	p^b^				
HHS after 1 week	31.9 (±14.2)	36.3 (±11.6)	38.4 (±10.7)	34.3 (±13.0)	36.9 (±11.3)	0.048	0.016	0.20	0.041	0.037	0.64
HHS after 6 weeks	68.7 (±13.5)	70.8 (±10.1)	70.2 (±11.7)	69.9 (±11.8)	70.6 (±10.5)	0.45	0.21	0.51	0.32	0.27	0.78
HHS after 6 months	85.6 (±13.4)	87.4 (±10.4)	88.1 (±9.6)	86.6 (±11.8)	87.6 (±10.1)	0.61	0.37	0.48	0.38	0.47	0.64
ODI after 1 week	55.1% (±16.8%)	51.2% (±16.4%)	48.0% (±12.4%)	53.0% (±16.7%)	50.4% (±15.5%)	0.15	0.072	0.19	0.077	0.15	0.45
ODI after 6 weeks	20.8% (±14.8%)	16.3% (±7.3%)	16.8% (±7.3%)	18.4% (±11.5%)	16.5% (±7.3%)	0.22	0.086	0.70	0.31	0.092	0.84
ODI after 6 months	9.5% (±12.8%)	6.4% (±8.5%)	3.0% (±5.0%)	7.8% (±10.7%)	5.5% (±7.8%)	0.011	0.040	0.0059	0.0039	0.22	0.022

K-W—Kruskal–Wallis test; p^a^—Kruskal–Wallis test result; p^b^—U Mann–Whitney test result; HHS—Harris Hip Score; ODI—Oswestry Disability Index.

**Table 4:** Association between *COMT* rs4818 genotype and the degree of disability in patients after THR.

Parameter	*COMT* rs4818										
	*CC* (*n* = 76)	*CG* (*n* = 88)	*GG* (*n* = 31)	*CC+CG* (*n* = 164)	*CG+GG* (*n* = 119)	K-W	*CC* vs. *CG+GG*	*CC+CG* vs. *GG*	*CC* vs. *GG*	*CC* vs. *CG*	*CG* vs. *GG*
	Mean (±SD)					p^a^	p^b^				
HHS after 1 week	31.9 (±14.1)	36.4 (±11.6)	38.4 (±10.7)	34.3 (±13.0)	36.9 (±11.4)	0.041	0.013	0.20	0.038	0.031	0.67
HHS after 6 weeks	68.8 (±13.4)	70.8 (±10.2)	70.2 (±11.7)	69.9 (±11.8)	70.6 (±10.6)	0.44	0.21	0.51	0.32	0.26	0.79
HHS after 6 months	85.4 (±13.4)	87.7 (±10.2)	88.1 (±9.6)	86.6 (±11.8)	87.8 (±10.0)	0.50	0.26	0.48	0.33	0.34	0.70
ODI after 1 week	55.2% (±16.7%)	51.1% (±16.5%)	48.0% (±12.4%)	53.0% (±16.7%)	50.3% (±15.6%)	0.13	0.061	0.19	0.072	0.13	0.47
ODI after 6 weeks	20.9% (±14.7%)	16.2% (±7.3%)	16.8% (±7.3%)	18.4% (±11.5%)	16.4% (±7.3%)	0.15	0.055	0.70	0.27	0.057	0.77
ODI after 6 months	9.8% (±12.9%)	6.1% (±8.2%)	3.0% (±5.0%)	7.8% (±10.7%)	5.3% (±7.6%)	0.008	0.022	0.0059	0.0031	0.14	0.027

K-W—Kruskal–Wallis test; p^a^—Kruskal–Wallis test result; p^b^—U Mann–Whitney test result; HHS—Harris Hip Score; ODI—Oswestry Disability Index.

**Table 5:** Association of *COMT* rs4680 genotype with the degree of disability in patients after THR.

Parameter	*COMT* rs4680										
	*AA* (*n* = 47)	*GA* (*n* = 101)	*GG* (*n* = 47)	*AA+GA* (*n* = 148)	*GA+GG* (*n* = 148)	K-W	*AA* vs. *GA+GG*	*AA+GA* vs. *GG*	*AA* vs. *GG*	*AA* vs. *GA*	*GA* vs. *GG*
	Mean (±SD)					p^a^	p^b^				
HHS after 1 week	32.7 (±14.7)	35.5 (±11.9)	35.9 (±12.2)	34.6 (±12.9)	35.7 (±12.0)	0.42	0.19	0.67	0.21	0.25	0.93
HHS after 6 weeks	70.1 (±12.2)	69.6 (±12.1)	70.4 (±10.7)	69.8 (±12.1)	69.9 (±11.7)	0.71	0.68	0.42	0.48	0.84	0.46
HHS after 6 months	84.4 (±14.8)	87.9 (±10.2)	87.0 (±10.2)	86.8 (±11.9)	87.6 (±10.2)	0.53	0.27	0.89	0.46	0.26	0.84
ODI after 1 week	55.3% (±16.6%)	51.5% (±16.6%)	50.6% (±14.5%)	52.7% (±16.7%)	51.2% (±15.9%)	0.36	0.16	0.57	0.19	0.21	0.95
ODI after 6 weeks	19.7% (±12.9%)	18.4% (±11.6%)	15.9% (±6.4%)	18.8% (±12.0%)	17.6% (±10.3%)	0.31	0.29	0.16	0.14	0.51	0.25
ODI after 6 months	9.9% (±12.5%)	7.3% (±10.2%)	3.7% (±5.7%)	8.1% (±11.0%)	6.2% (±9.2%)	0.006	0.029	0.0038	0.0018	0.18	0.020

K-W—Kruskal–Wallis test; p^a^—Kruskal–Wallis test result; p^b^—U Mann–Whitney test result; HHS—Harris Hip Score; ODI—Oswestry Disability Index.

**Table 6:** The impact of the number of rs4818 *COMT G* alleles on the degree of hip functional performance as measured by the HHS one week after hip arthroplasty in a multivariate linear regression model.

Parameter	Coefficient in the Regression Equation	Beta (95%CI)	*p*
Age [years]	+0.010	+0.009 (−0.132–+0.150)	0.90
Sex [male vs. female]	+0.83	+0.031 (−0.109–+0.172)	0.66
*COMT* rs4818 (number of G alleles)	+3.53	+0.196 (0.056–+0.336)	0.0064

**Table 7:** Impact of the number of rs4818 *COMT G* alleles on the degree of disability as measured by the ODI six months after THR in a multivariate linear regression model.

Parameter	Coefficient in the Regression Equation	Beta (95%CI)	*p*
Age [years]	+0.078	+0.084 (−0.055–+0.223)	0.23
Sex [male vs. female]	−0.65	−0.031 (−0.169–+0.108)	0.66
*COMT* rs4818 (number of *G* alleles)	−3.49	−0.241 (−0.378–−0.103)	0.00070

**Table 8:** Impact of the number of rs4818 *G* alleles on the change in degree of disability between one week and six months after THR, as measured by the ODI in a multivariate linear regression model.

Parameter	Coefficient in the Regression Equation	Beta (95%CI)	*p*
Age [years]	+0.29	+0.123 (−0.017–+0.264)	0.085
Sex [male vs. female]	+2.32	−0.043 (−0.097–+0.183)	0.54
*COMT* rs4818 (number of G alleles)	−5.83	−0.160 (−0.300–−0.020)	0.025

## Data Availability

Data available on request due to restrictions privacy.
